# Biomechanics of the Cornea Evaluated by Spectral Analysis of Waveforms from Ocular Response Analyzer and Corvis-ST

**DOI:** 10.1371/journal.pone.0097591

**Published:** 2014-08-27

**Authors:** Sushma Tejwani, Rohit Shetty, Mathew Kurien, Shoruba Dinakaran, Arkasubhra Ghosh, Abhijit Sinha Roy

**Affiliations:** 1 Glaucoma Service, Narayana Nethralaya, Bangalore, Karnataka, India; 2 Refractive Surgery, Narayana Nethralaya, Bangalore, Karnataka, India; 3 Cataract Surgery, Narayana Nethralaya, Bangalore, Karnataka, India; 4 Gene, Repair and Regeneration in Ocular Workstation (GROW research lab), Narayana Nethralaya, Bangalore, Karnataka, India; 5 Imaging, Biomechanics and Mathematical Modeling Solutions (IBMS) lab, Narayana Nethralaya, Bangalore, Karnataka, India; University of Missouri-Columbia, United States of America

## Abstract

**Purpose:**

In this study, spectral analysis of the deformation signal from Corvis-ST (CoST) and reflected light intensity from ocular response analyzer (ORA) was performed to evaluate biomechanical concordance with each other.

**Methods:**

The study was non-interventional, observational, cross-sectional and involved 188 eyes from 94 normal subjects. Three measurements were made on each eye with ORA and CoST each and then averaged for each device. The deformation signal from CoST and reflected light intensity (applanation) signal from ORA was compiled for all the eyes. The ORA signal was inverted about a line joining the two applanation peaks. All the signals were analyzed with Fourier series. The area under the signal curves (AUC), root mean square (RMS) of all the harmonics, lower order (LO included 1^st^ and 2^nd^ order harmonic), higher order (HO up to 6^th^ harmonic), CoST deformation amplitude (DA), corneal hysteresis (CH) and corneal resistance factor (CRF) were analyzed.

**Results:**

The device variables and those calculated by Fourier transform were statistically significantly different between CoST and ORA. These variables also differed between the eyes of the same subject. There was also statistically significant influence of eyes (left vs. right) on the differences in a sub-set of RMS variables only. CH and CRF differed statistically significantly between the eyes of subject (p<0.001) but not DA (p = 0.65).

**Conclusions:**

CoST was statistically significantly different from ORA. CoST may be useful in delineating true biomechanical differences between the eyes of a subject as it reports deformation.

## Introduction

The cornea is responsible for nearly 80% of the total refractive power of the human eye. To achieve good quality vision, the cornea has an intricate structure of collagen fibers arranged in lamellae and interwoven with a cellular matrix to achieve and maintain a specific curvature [1]. Thus, the biomechanical status of the cornea plays a key role in maintaining quality vision [2], [3]. There are several refractive procedures, e.g., Laser-Assisted in situ keratomileusis (LASIK), photorefractive keratectomy (PRK), small incision lenticule extraction (SMILE), where the cornea may have some biomechanical changes after the treatment [4], [5]. Similarly, small incisions in cataract surgery are known to induce unwanted astigmatism in the cornea [6]. There are also several corneal disorders, e.g., keratoconus, pellucid marginal degeneration, where the native lamellar structure is disrupted and the cornea needs transplantation or collagen crosslinking [7], [8], [9]. In all these procedures, the pre-operative biomechanical status, e.g., elastic modulus, of the cornea is not known.

Present clinical evaluation of biomechanics of the cornea is limited to qualitative analysis only as direct measures of moduli are not available. There are presently two clinical devices, the Ocular Response Analyzer or ORA (Reichert Inc., USA) [10] and Corvis-ST or CoST (Oculus Optikgeräte GmbH, Germany) that are capable of measuring the dynamic mechanical properties of the cornea [11]. Both ORA and CoST apply an air-puff on the anterior surface of cornea and cause the cornea to deform. The air-puff technique is commonly used for intraocular pressure measurement. The ORA measures the intensity of the reflected infrared light from the deforming corneal surface and reports several indices for diagnosis [10]. Among them, corneal hysteresis (CH) and corneal resistance factor (CRF) have been investigated extensively in different patient groups [12], [13], [14]. These measures provide qualitative information about the biomechanical state of the cornea and additional waveform analysis has been investigated [15], [16], [17]. CoST also uses an air-puff to deform the cornea. It uses Scheimpflug imaging and a high-speed camera to record images of a cross-section (the horizontal meridian) of the cornea as it deforms. This enables CoST to provide quantitative information, e.g., displacement of the cornea apex, which may be more relevant for evaluation of corneal biomechanics.

Theoretically, both ORA and CoST measure corneal deformation. However, the ORA output is the signal intensity of the reflected infrared light represented in scalar arbitrary units, i.e., it does not account for direction of corneal motion. On the other hand, CoST reports the corneal displacement (magnitude and direction of motion). Recent studies have shown that spectral analysis of ocular waveforms can provide useful information pertaining to clinical diagnosis of conditions [18], [19]. In this study, spectral analyses of the waveforms reported by ORA and CoST in a cohort of patients with *apparent* normal corneas were conducted. In this study, the biomechanical differences between the devices were evaluated. Further, the influence of fellow eye on differences between the devices was also evaluated. The waveforms were normalized with their respective peak values such that the transformed values of the data points ranged from 0 to 1. Spectral analysis converted these waves into frequency components (harmonics), which were used to define several diagnostic indices.

## Methods

The study was a prospective, observational, cross-sectional study performed in Narayana Nethralaya, India. The study was approved by the institutional review board at the Narayana Nethralaya. Informed written consent was obtained from all the subjects and the study adhered to the tenets of the Declaration of Helsinki. Both eyes of 94 subjects were evaluated with the ORA and CoST. The inclusion criteria for the study were age between 18–80 years of age, were either emmetropes or had refractive error between −5D and +3D. Subjects with cataract and pseudophakes that were operated more than a year ago were included. This was done to eliminate any factors that would have affected the corneal biomechanical properties significantly. Similarly subjects with altered corneal biomechanics, e.g., keratoconus, any other cornea problems, e.g., pellucid marginal corneal degeneration, prior refractive surgery, prior cornea surgery, prior retina surgery, cataract surgery done within last one year, aphakia, refractive error between −5.0D and +3.0D, and astigmatism greater than +3.0D were not included in the study. The screening of the patients was performed based on history, retinoscopy [20], refraction (both objective and subjective) and detailed clinical evaluation by experienced ophthalmologists. Any suspects on clinical grounds underwent corneal topography but all the patients were not subjected to topography to rule out the subclinical disease.

### (a) Spectral Analysis of Waveforms from ORA and CoST

**Figure 1:**
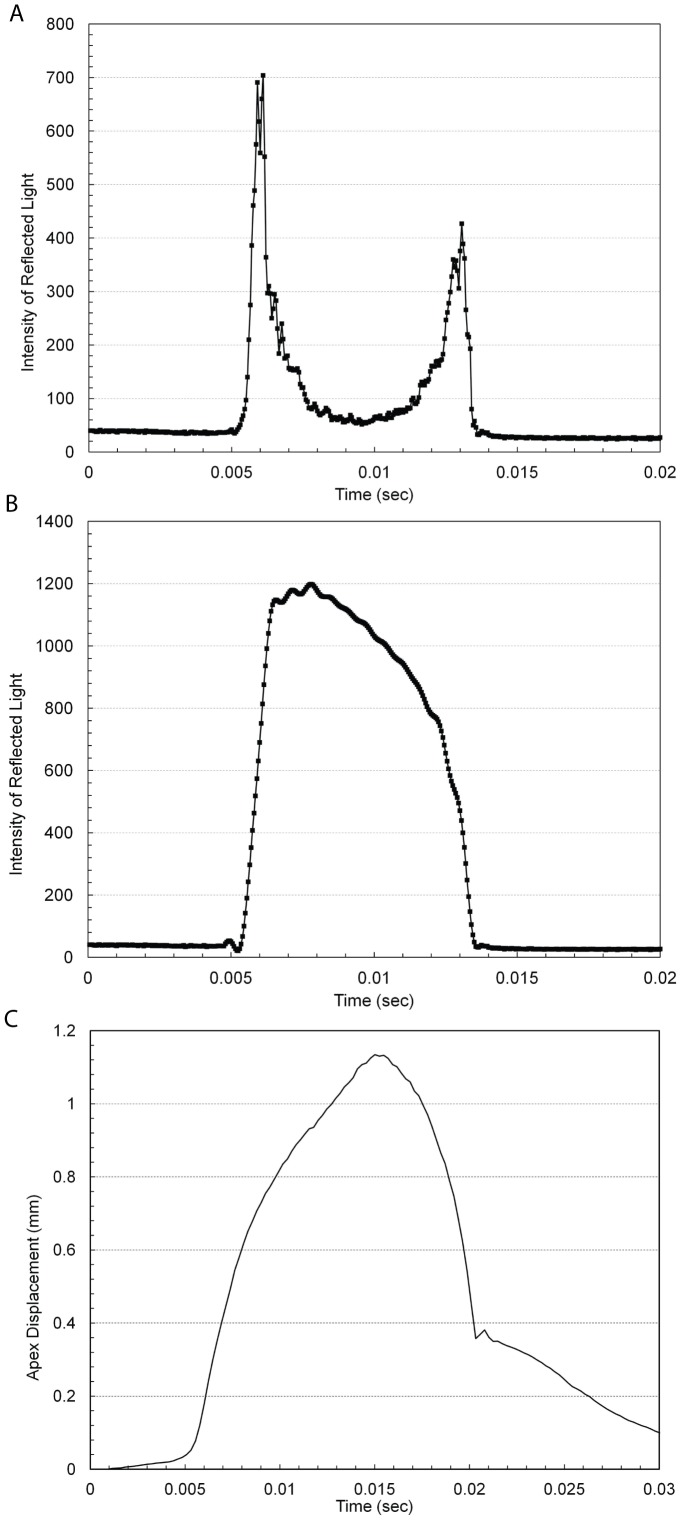
Waveforms reported by Ocular response analyzer (ORA) and Corvis-ST: (a) Example of ORA waveform. The two peaks represent the instant the cornea becomes flat during the forward and backward motion of the cornea; (b) the same OA waveform now inverted about the straight line joining the two peaks in figure 1a; (c) Example of the apex displacement in mm measured by CoST.

The ORA allowed export of the signal intensity waveform (applanation curve) as a comma separated value file. Figure 1a shows a sample measurement by the ORA. The two peaks were the signal intensities at which the cornea becomes flat, the 1^st^ during increasing pressure and the 2^nd^ during decreasing pressure (see figure 1a). The signal intensity decreased between the two peaks because the angle of reflection changes during deformation but the position of beam detector remains fixed. Thus, the region of the waveform between the two peaks was not as accurate as the data collected before the 1st peak and after the 2nd peak. However, some studies have shown statistically significant differences between normal and treated/disease patients in the region between the two peaks of the waveform [15], [16], [17]. Since the intensity was reported as a scalar and does not represent the direction of motion of the cornea during deformation, the region between the two peaks was inverted about a straight line joining the two peaks. Inversion was achieved by adding twice the linear distance of the data point along the y-axis from the straight line. Inversion of the waveform in figure 1a is shown in figure 1b. After inversion, Fourier transform (FT) of the waveform was performed. FT subdivided the wave into its harmonics, each quantified by a phase and magnitude. The magnitude of each harmonic was computed as follows:
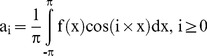
(1)

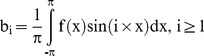
(2)where a_i_ and b_i_ were the amplitudes of the cosine and sine components (also called harmonics), x was the non-dimensional time ( = 2π×(t/T−0.5); t and T were the current and cycle time, respectively), and f(x) was the waveform derived after inversion. i represented the i^th^ harmonic of the FT. It was assumed that the cycle time of the ORA was the same as the duration of the applanation test. Similarly, the a_i_'s and b_i_'s were computed for the waveform derived from CoST except that no inversion of the CoST waveform was necessary. The FT of the inverted ORA and CoST waveforms are shown in figures 1b and c, respectively. The FT was performed using a custom script written in Python (v.2.7). CoST waveform was extracted from the screen capture of the device computer monitor using Image J (v1.46).

### (b) Diagnostic Variables Tested

The area under the curves was computed (AUC_ORA_ and AUC_CoST_) using a 2^nd^ order accurate numerical integration scheme. However, the ORA and CoST waveform was in arbitrary unit and mm, respectively. To allow comparison of the harmonics of the two curves, each curve was normalized to a range between 0 and 1 by dividing with its' respective peak data point and then the FT was repeated to obtain a_i_ and b_i_ for the normalized curves. The FT was performed up to order (n being the total number of harmonics) 31. Thus, the AUC's, a_i_'s and b_i_'s were computed using the normalized data points. The following additional variables were defined for statistical comparison of the FT's of each subject:
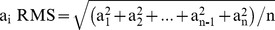
(3)

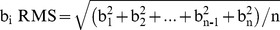
(4)

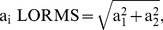
(5)

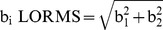
(6)

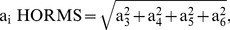
(7)

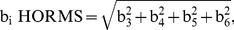
(8)RMS was the root mean square. LORMS and HORMS were defined as lower and higher order (or harmonic) root mean square, respectively and were a subset of the corresponding RMS variables. This form of RMS was chosen similar to RMS used in characterizing the corneal and ocular wavefront with Zernike polynomials prior to any refractive surgery. Other variables that were analyzed were corneal hysteresis (CH), corneal resistance factor (CRF) from ORA, and deformation amplitude (DA) from CoST.

### (c) Statistical Methods

The normality of the data was evaluated with Chi-square test. Two-way (i.e., difference between devices was the primary factor and effect of the eye measured (left or right) on differences between the devices was the secondary factor) analysis of variance (2-way ANOVA) was used. If 2-way ANOVA resulted in any statistical significance, post-hoc comparison of the effect of factors on group means was performed. All p-values reported from two-way analysis were Bonferroni corrected. 1-way analysis of variance was used to compare differences between measured CH, CRF and DA of the left and right eyes of subjects. Linear regression analysis between the variables and age of the subjects was performed. P-value less than 0.05 was considered statistically significant. The statistical analyses were performed in MedCalc v.12.7.7 (MedCalc Inc., Belgium).

## Results

**Figure 2:**
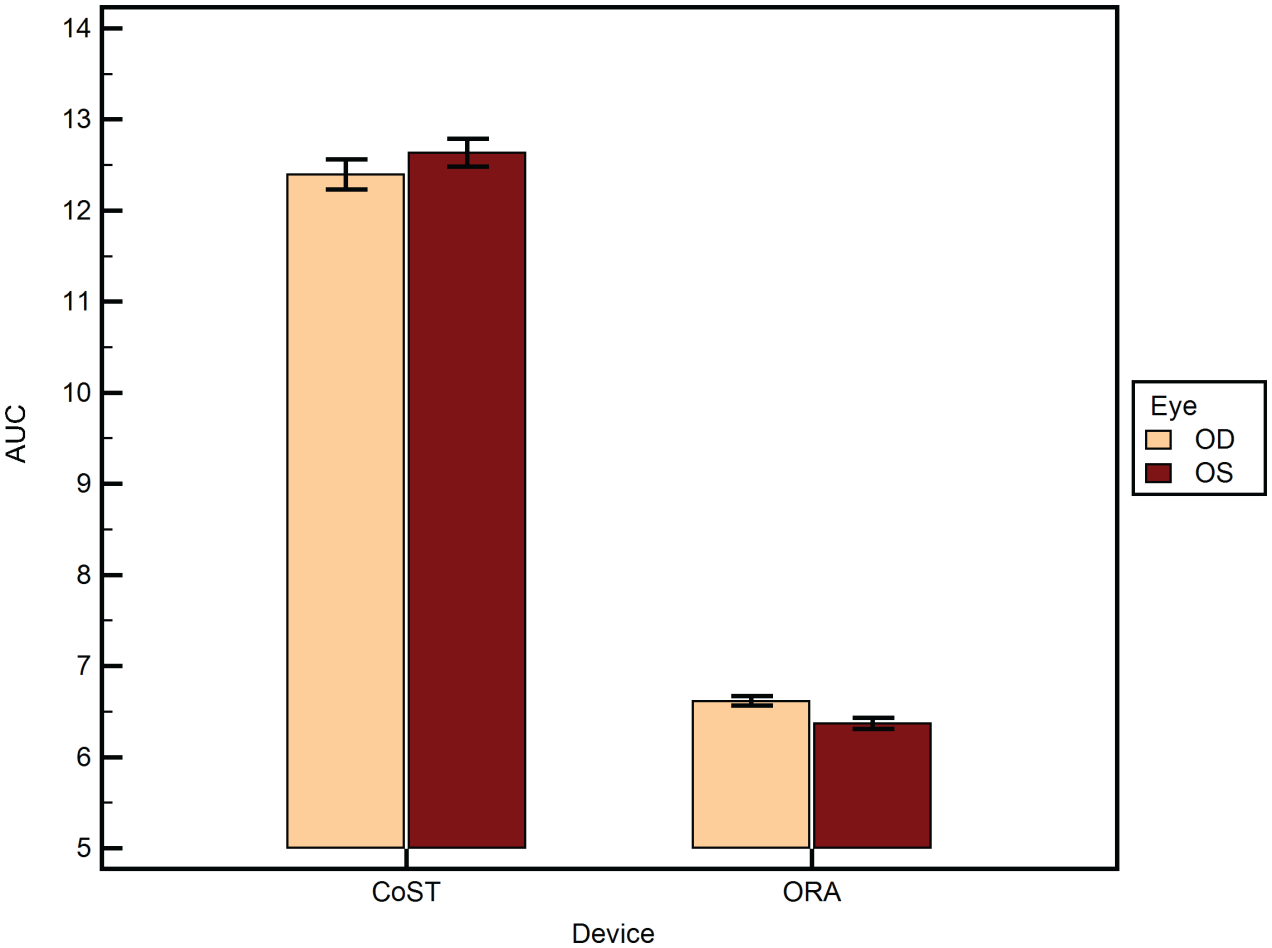
Area under the curve (AUC) from ORA and CoST segregated by the fellow eyes (OD = right and OS = left eye). The mean±SEM is plotted. SEM is the standard error of the mean.

**Figure 3:**
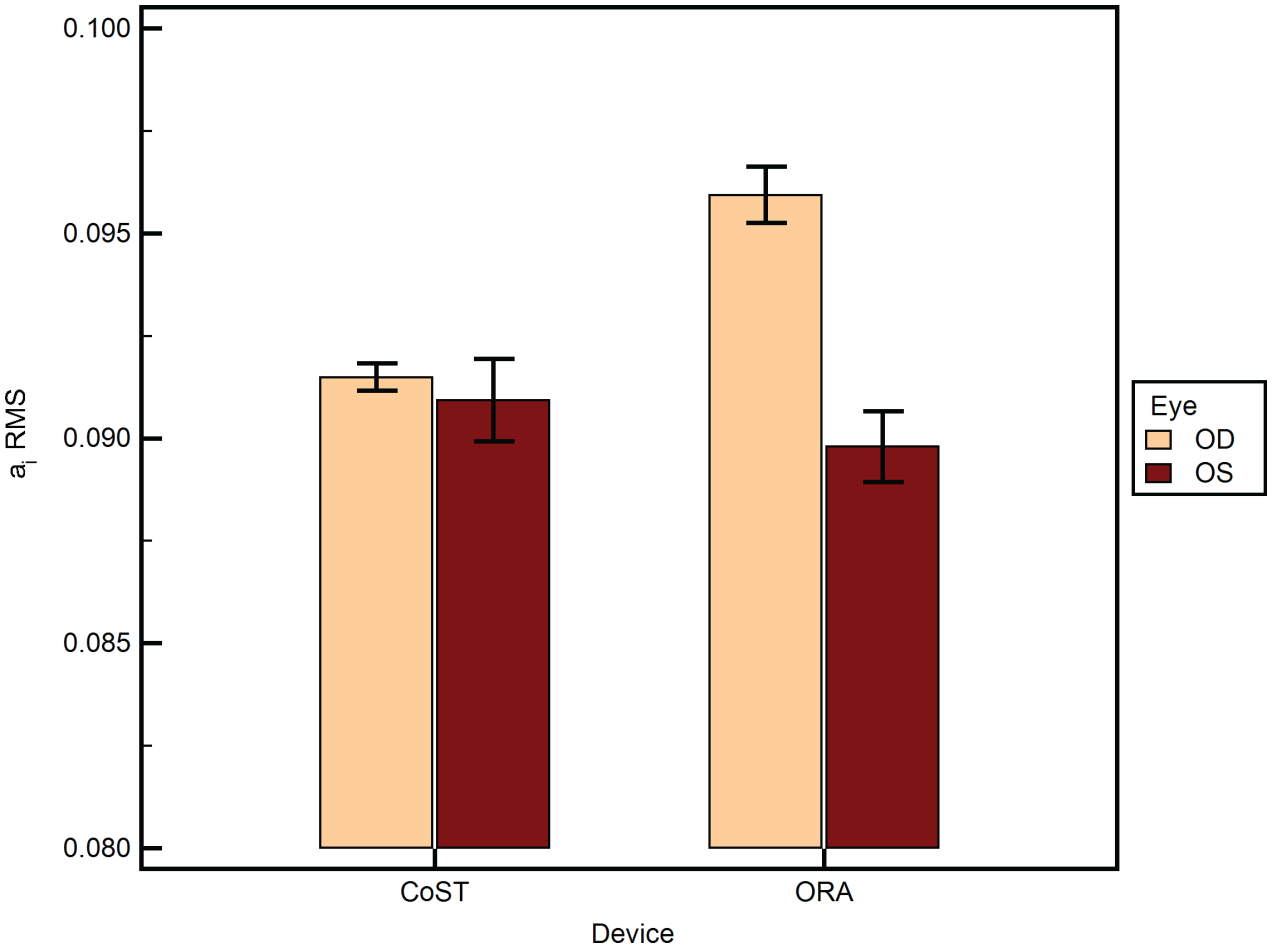
a_i_ RMS (root mean square) under the curve (AUC) from ORA and CoST segregated by the fellow eyes (OD = right and OS = left eye). The means±SEM are plotted. SEM is the standard error of the mean.

**Figure 4:**
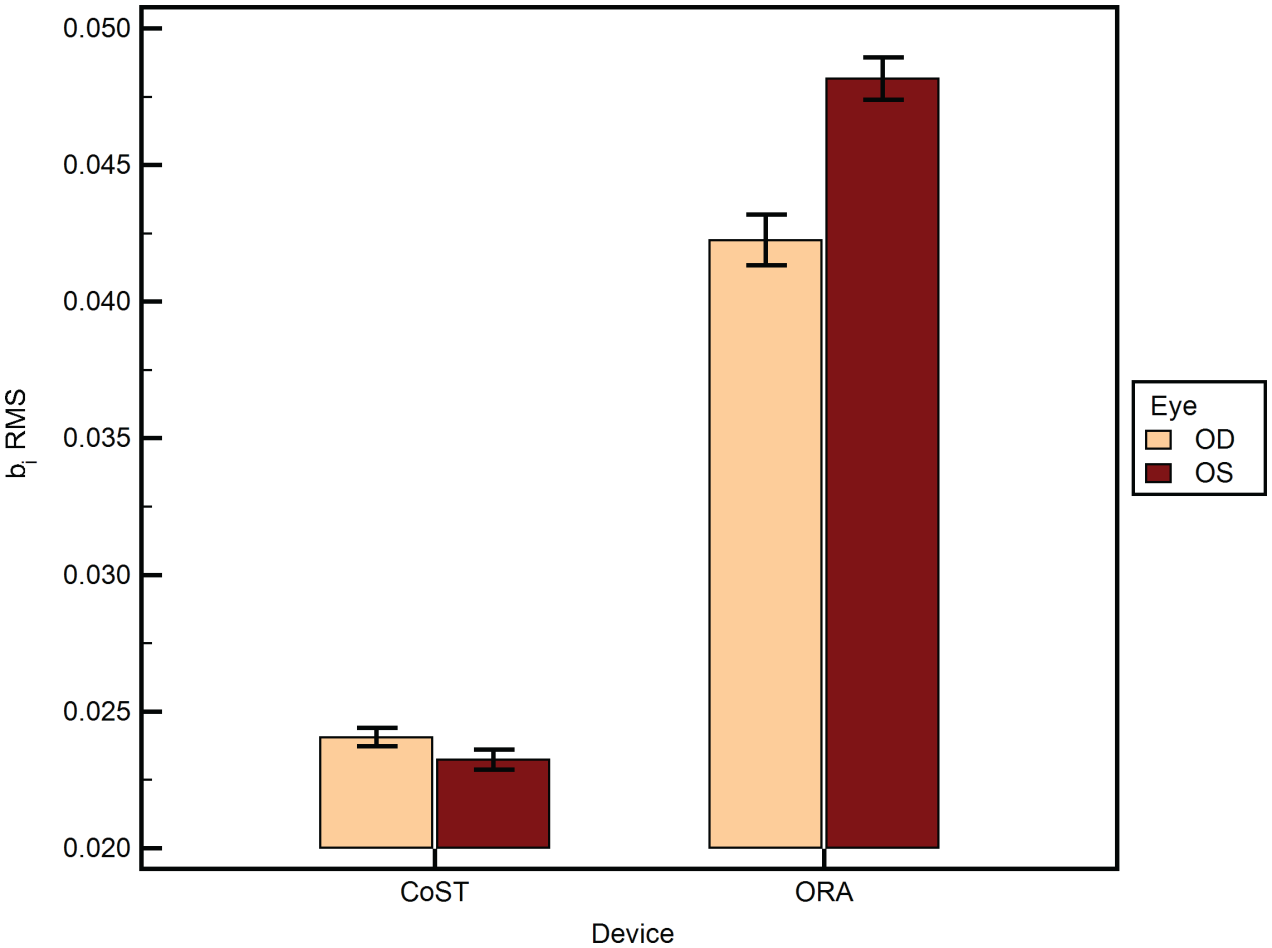
b_i_ RMS (root mean square) under the curve (AUC) from ORA and CoST segregated by the fellow eyes (OD = right and OS = left eye). The means±SEM are plotted. SEM is the standard error of the mean.

**Figure 5:**
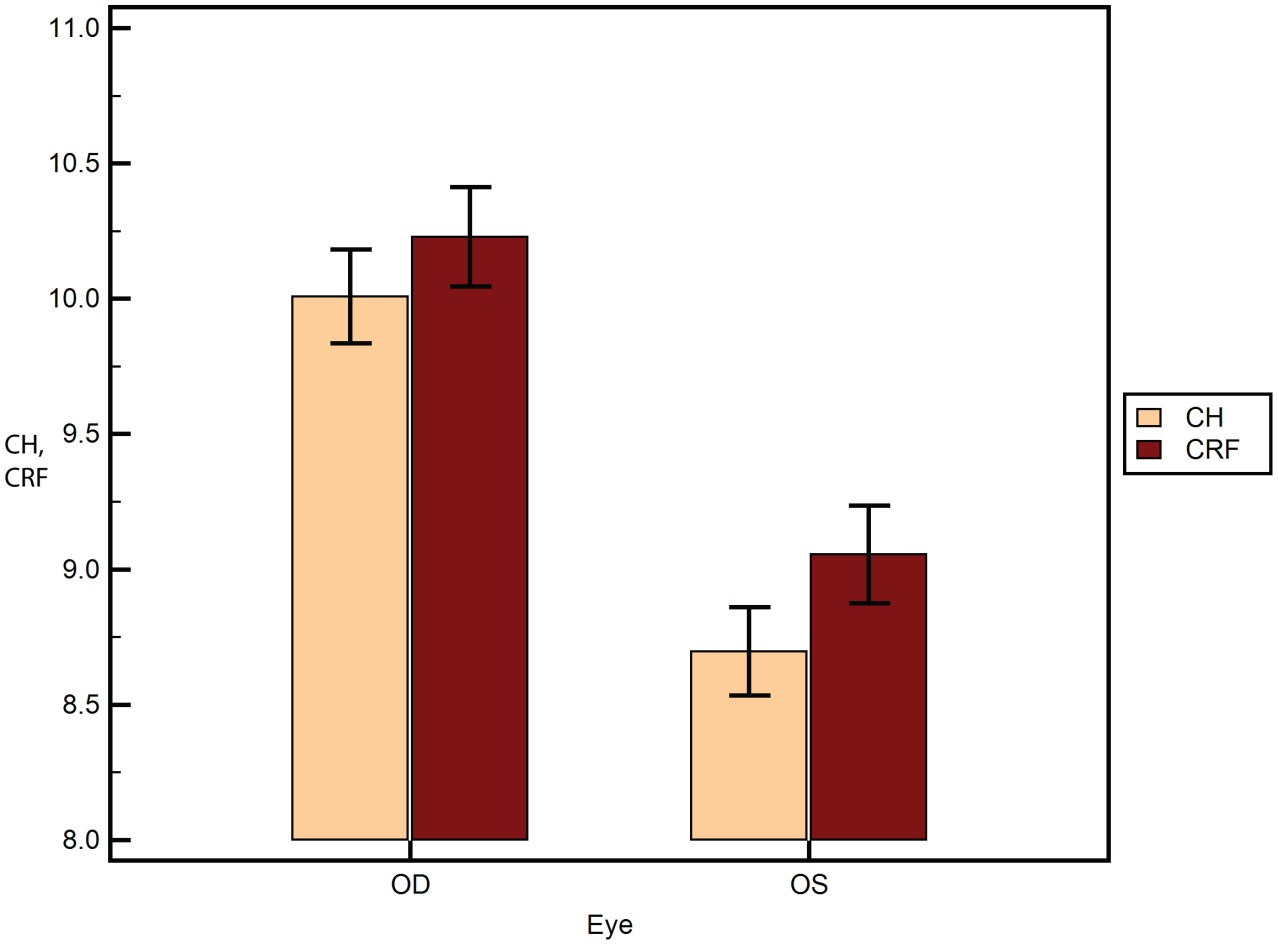
Corneal hysteresis (CH) and corneal resistance factor (CRF) between the fellow eyes of the subjects. The means±SEM are plotted. SEM is the standard error of the mean.

**Figure 6:**
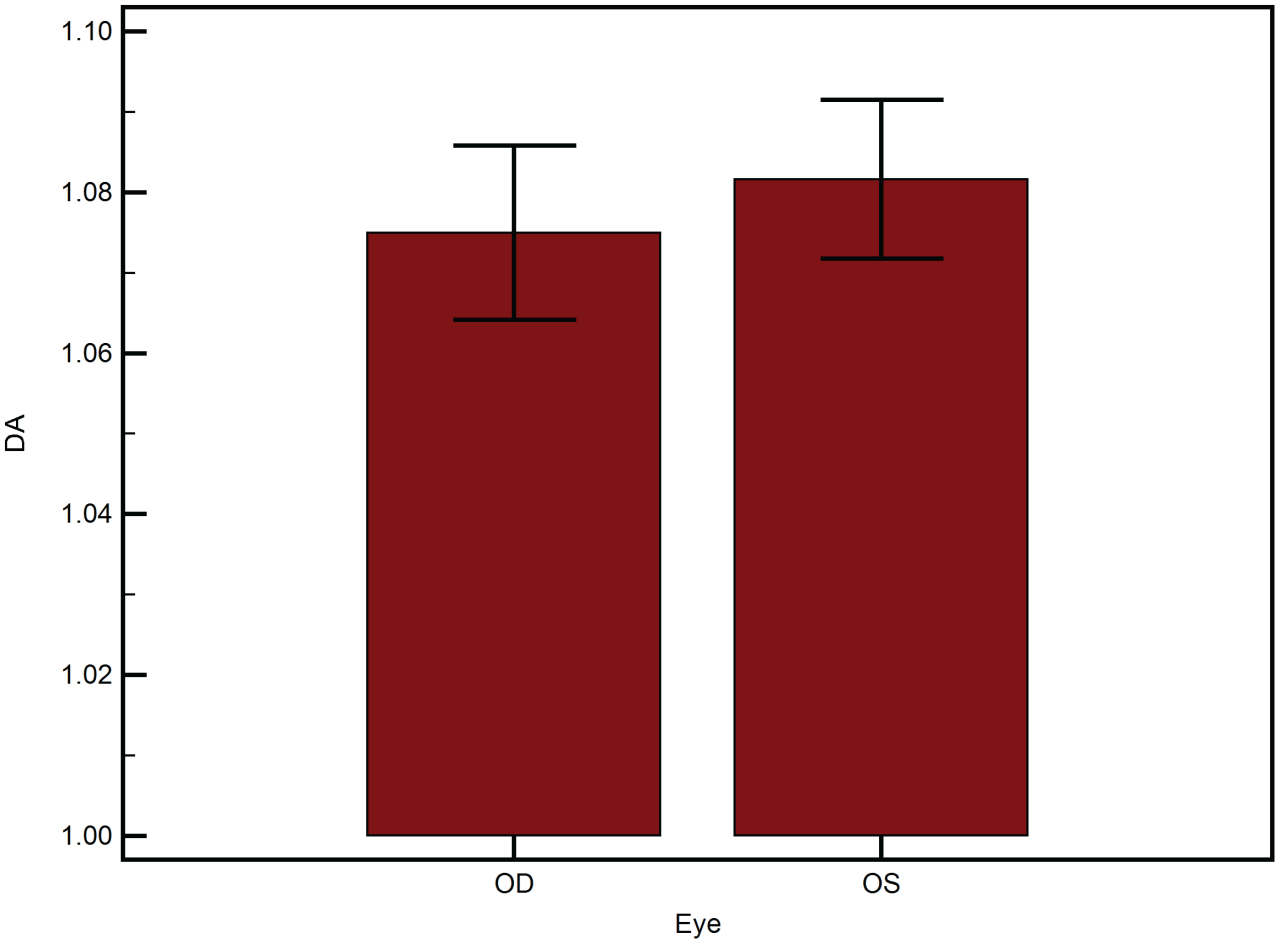
Deformation amplitude (DA) between the fellow eyes of the subjects. The means±SEM are plotted. SEM is the standard error of the mean.

A total of 188 eyes of 94 subjects were analyzed. Table 1 lists the mean of the different variables as mean±SEM, where SEM is the standard error of mean. The mean age was 35.7±1.33 years. The male to female ratio was 0.85. There was no statistically significant difference between the central corneal thickness of the left and right eyes of the subjects (p = 0.79). 2-way ANOVA of AUC was statistically significantly different between the devices (figure 2) (p<0.001). However, AUC did not differ between the eyes of the same subject (p>0.05). There was no influence of eye measured (left or right) on the difference between the two devices (p>0.05). a_i_ RMS (figure 3) was statistically significantly different between the devices (p = 0.03) and between the eyes (p<0.001). Further, the eye measured (left or right) had a statistically significant influence on the difference between the two devices (p<0.001). Similarly, b_i_ RMS (figure 4) was statistically significantly different between the devices (p = 0.03), between the eyes (p<0.001), and the eye measured (left or right) had a statistically significant influence on the difference between the two devices (p<0.001). A similar statistical inference was made from computed values of a_i_ LORMS and b_i_ LORMS. However, a_i_ HORMS was and was not statistically significantly different between the two devices (p<0.001) and between the eyes (p = 0.07), respectively. Further, the eye measured (left or right) did not influence the difference between the two devices (p = 0.17). Similarly, b_i_ HORMS was and was not statistically significantly different between the two devices (p<0.001) and between the eyes (p = 0.47), respectively. Further, the eye measured (left or right) did not influence the difference between the two devices (p = 0.22). CH and CRF (figure 5) differed statistically significantly between the eyes of subjects (p<0.001) but not DA (figure 6) (p = 0.65). Linear regression analysis between age and the all the variables was not statistically significant (p>0.05), i.e., slope was not different from zero.

**Table 1:** Mean and SEM of the variables, where SEM is the standard error of the mean.

	CoST				ORA							
OD	AUC_CoST_	a_i_ RMS	b_i_ RMS	DA	AUC_ORA_	a_i_ RMS	b_i_ RMS	IOP_g_	IOP_cc_	CRF	CH	Age
Median	12.4	0.091	0.024	1.08	6.62	0.096	0.042	16	16.9	10.23	10.0	35.7
SEM	0.16	0.0003	0.0003	0.01	0.051	0.0007	0.0009	0.47	0.47	0.18	0.17	1.33
OS												
Median	12.63	0.091	0.023	1.08	6.37	0.09	0.048	15.8	18.1	9.06	8.69	35.7
SEM	0.15	0.001	0.0004	0.01	0.063	0.0009	0.008	0.53	0.53	0.18	0.163	1.33

AUC_CoST_ (Area under CoST curve) has unit of mm-msec. AUC_ORA_ (Area under the ORA curve) has unit of a.u.-msec. a_i_ and b_i_ root mean square (RMS) has unit of mm for CoST. a_i_ and b_i_ root mean square (RMS) has unit of a.u. for CoST. IOP_g_ (ORA Goldmann correlated intraocular pressure), IOP_cc_ (ORA Corneal compensated intraocular pressure), CH (Corneal hysteresis) and CRF (Corneal resistance factor) have unit of mmHg. Deformation amplitude (DA) is in mm.

**Figure 7:**
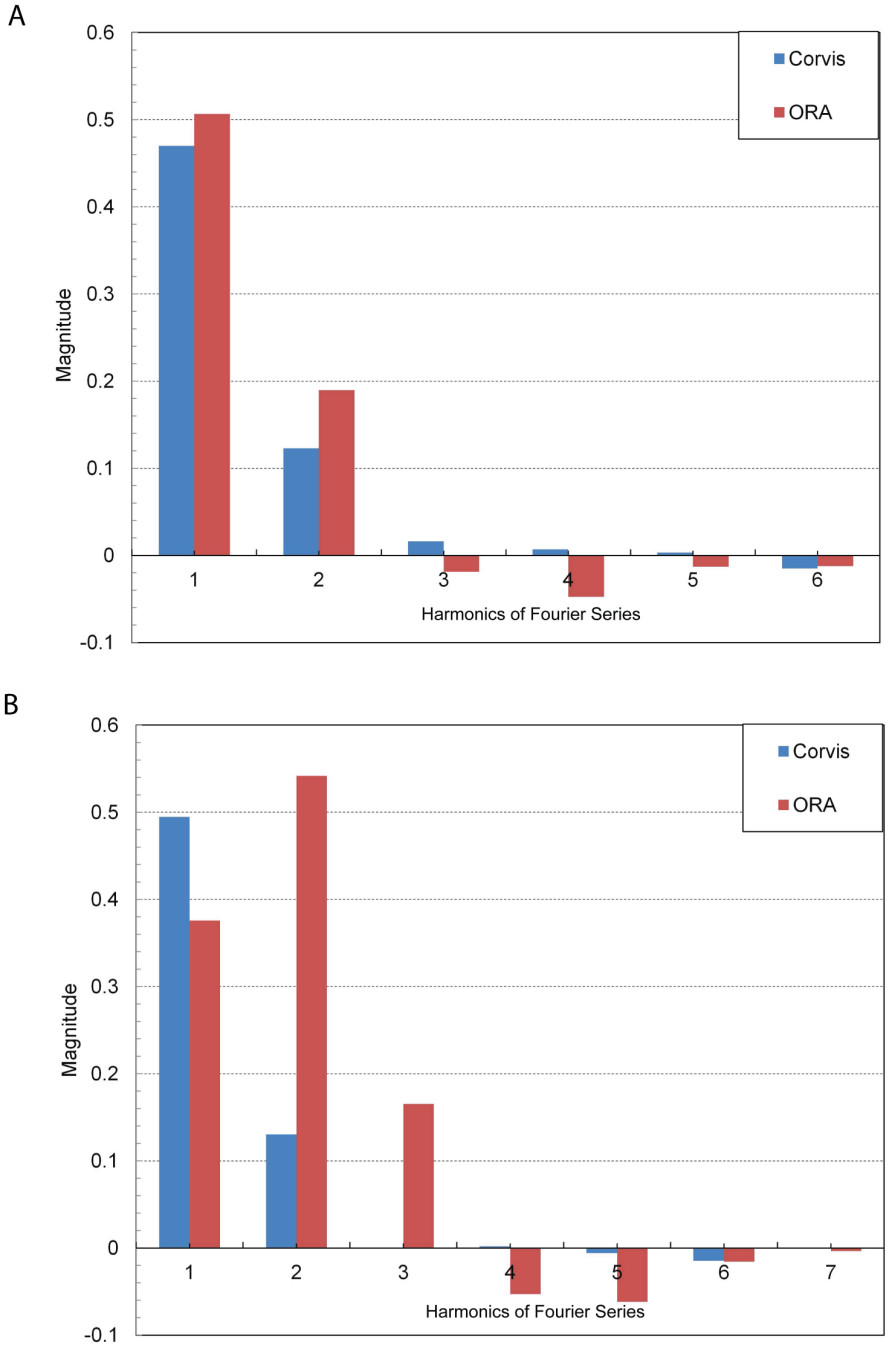
Cosine harmonics from 1^st^ to 6^th^ order in a subject with CCT equal to: (a) 503 micron; (b) 525 micron. The numbers on the horizontal axis represent the order of the harmonics.

**Figure 8:**
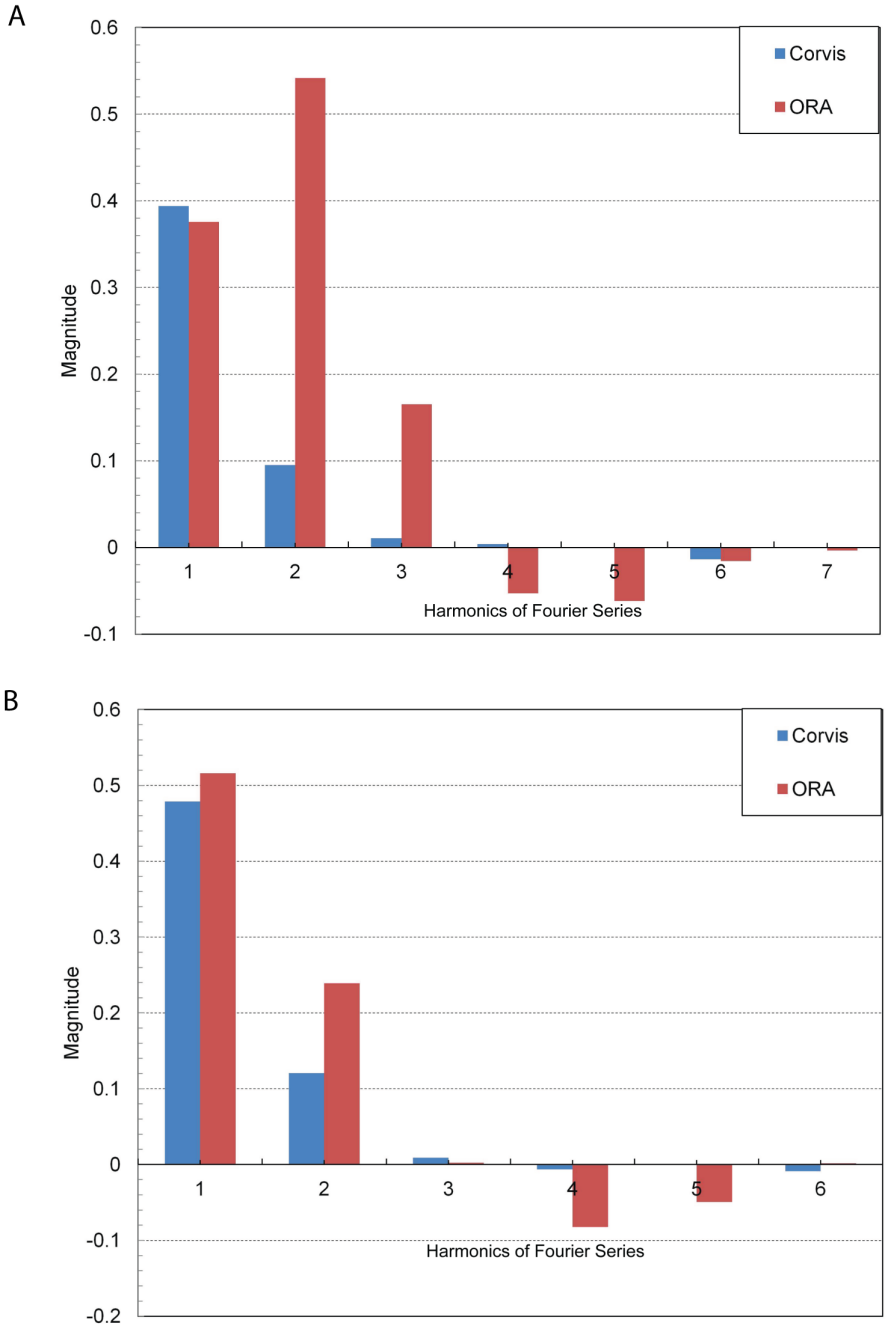
Sine harmonics from 1^st^ to 6^th^ order in a subject with CCT equal to: (a) 553 micron; (b) 581 micron. The numbers on the horizontal axis represent the order of the harmonics.

A comparison of magnitudes of cosine harmonic (a_i_) up to 6^th^ order in subjects with CCT ranging from 505 to 581 microns has been shown in figures 2 and 3. In figure 7a, the subject had a CCT of 503 micron, AUC_CoST_ was 11.6 mm-msec and DA was 1.01 mm. The 1st and 2nd harmonic had the largest magnitudes compared to other harmonics in both ORA and CoST waveforms. ORA harmonics (3^rd^ and higher) were larger than the corresponding values for CoST. In figure 7b, the subject had a CCT of 525 micron, AUC_CoST_ was 12.52 mm-msec and DA was 1.12 mm. The discrepancy between ORA and CoST was greatest for the 2nd harmonic. In figure 8a, the subject had a CCT of 553 micron, AUC_CoST_ was 9.97 mm-msec and DA was 0.98 mm. The 2nd harmonic showed the most discrepancy between the two devices once again. In figure 8b, the subject had a CCT of 581 micron, AUC_CoST_ was 11.32 mm-msec and DA was 0.97 mm. The magnitude of the harmonics was similar to the subject described in figure 2a. Thus, while the FT distinct different differences between subjects based on harmonics, there were no significant trends between AUC, DA and CCT.

## Discussion

The study demonstrated that spectral components of ORA and CoST waveforms can be computed and the harmonics provided additional information about the biomechanical status of the cornea. Waveform analysis of the ORA wave has been performed in both normal and disease corneas [17], [18], [21], [22]. In a recent study, the ORA pressure was plotted against the applanation signal and then the region of the curve that lay in the “trough” of the ORA signal was inverted [21]. The resulting curve was remarkably similar to curves obtained from viscoelastic measurements of soft tissues on the bench [21]. As per viscoelastic theory, the area enclosed by a stress vs. strain curve (obtained from *ex vivo* testing of tissue) is known as hysteresis. It is the energy dissipated in the tissue when external forces (air-puff) cause deformation of the tissue and this dissipated energy cannot be recovered when the forces are removed. In both CoST and ORA, the applanation pressure can be considered analogous to stress. In CoST, deformation was a measure of strain. In ORA, the reflected light intensity was a measure of strain. However, CoST does not report the pressure signal and therefore, only the deformation signal from CoST and the inverted applanation signal from ORA were compared. The area enclosed by these curves may provide useful information about the viscoelastic status of the cornea, e.g., larger area may correspond to higher viscosity and vice versa.

In our study, the part of the ORA waveform was inverted to make it similar to the CoST waveform. 2-way ANOVA showed statistically significant differences between the devices and between the eyes in most variables. In a few variables (RMS and LORMS variables), there was statistically significant influence of the measured eye on the differences between the devices. Similarly, CH and CRF were statistically significantly different between the left and right eye and not DA and it is generally accepted that the eyes of a subject are biomechanically similar, if they don't have any disease. From a biomechanical perspective, there are structural differences in layout of collagen distribution and orientation between the left and right eye [23]. In contrast, visual parameters, e.g., sphere, cylinder, axis, aberrations, do not appear to correlate between the fellow eyes [24], [25]. In this study, fellow eye differences were evident in a few variables but not all. Individual harmonics (a_i_'s and b_i_'s) also differed between the devices and between the eyes. This study cannot establish true differences between the biomechanical differences between the eyes of a patient for lack of a gold standard. However, ORA and CoST are the only clinical devices available and there is potential that CoST may be useful in quantification of biomechanics between the eyes since it reports deformation, which is a fundamental parameter to quantify biomechanics.

The change in CH and CRF reported by ORA in normal, disease and post-treatment corneas has been studied. In post-refractive patients, e.g., LASIK, both CH and CRF decreased after procedure. A combination of thickness reduction and change in the viscoelastic properties of the cornea may be responsible for the decrease in CH and CRF [13], [15], [16], [17]. A few studies had looked at features of the ORA waveform that may have more sensitivity to biomechanical changes in the cornea [15], [17], [18], [21]. These studies have highlighted salient features of the ORA waveform that could be useful for diagnosis of disease, e.g., a wider area under 1st or 2nd peak indicated a weaker cornea as the weaker cornea deformed slowly [17], [18]. Spectral analysis of the ocular pulse amplitude reported by dynamic contour tonometry had shown statistical differences between subjects with different types of glaucoma [19], [26]. Statistically significant differences were found between the harmonics of patients with primary open angle glaucoma and normal tension glaucoma, which may lead to newer diagnostic measures for identifying glaucoma suspects [19]. Age was not significantly correlated with any of the variables in this study and in past studies with other variables [27], [28]. This lack of correlation may be due to the diverse ethnic population and varying environmental conditions in India.

Spectral analysis of ocular wavefront has been commonly used in refractive surgery. Zernike polynomials are used to reconstruct the wavefront measured by an aberrometer. The magnitudes of these polynomials has formed the basis for wavefront guided surgery, topography guided surgery in highly aberrated corneas [2], [29]. This study demonstrated a similar form of analysis of the corneal deformation signal with RMS terms so as to identity *suspect* corneas before they are recommended for any corneal surgery. With this method, there may be a possibility to link visual outcomes such as corneal wavefront Zernike magnitudes after surgery to changes in corneal deformation harmonics, e.g., compare spectral analysis of deformation to corneal wavefront zernikes in crosslinked subjects. In keratoconic corneas, aberrations are higher and the Zernike terms also increase in magnitude. Similarly in disease corneas, the ORA and CoST waveforms had more undulations [21] and the Fourier harmonics could be expected to increase in magnitude. Spectral analysis of the deformation curve may also perform better than analyzing distinct features of the signal, e.g., slopes, inflection points.

The air-puff technique also suffers from limitations that may impact accurate determination of the biomechanical status of the cornea. Firstly, CoST recorded only 2-D cross-section images of the cornea. In disease (e.g. keratoconus) or treated (e.g. PRK) corneas, there may be lateral (out-of plane) motion of the cornea, which may not be captured by CoST. Secondly, air-puff technique caused the whole globe to move back when the air pulse impacted the cornea. Thus, the deformation signal and intensity of reflect wave from the anterior corneal surface were affected by it. In a recent study, the impact of orbital muscles was found to have a bigger impact on deformation history than the sclera [30]. Therefore, future studies need to focus on delineating the whole globe motion from the corneal deformation to improve the accuracy of the spectral analysis and to assess changes in the harmonics in different diseases and treatments. Another limitation was that the spectral analysis was performed on waveforms obtained from a single perturbation of the cornea and a complete, repeated deformation cycle was not obtained from either device. Therefore, some amount of spectral leakage can occur from Fourier transform. In spectral analysis of ocular pressure pulse wave, several continuous waveforms are available but not in ORA and CoST [19], [21], [22], [27], [28]. This limitation may be overcome to some extent in CoST, if the complete deformation history, i.e., corneal DA reached to zero, was provided. However, it was not possible to obtain multiple periods from either device as only one air-puff can be generated by each device in one measurement. Despite the limitation, the present Fourier transform was conducted with the basic assumption that unique features of the corneal deformation characterized by the harmonics obtained from ORA and CoST may be linked, e.g., whether increased a_i_ LORMS of subject cornea, when measured by ORA also implied increased a_i_ LORMS of the same eye when measured by CoST. From this study, it was evident that the two devices did not evaluate a given cornea similarly.
